# Dysregulated Pyrimidine Biosynthesis Contributes to 5-FU Resistance in SCLC Patient-Derived Organoids but Response to a Novel Polymeric Fluoropyrimidine, CF10

**DOI:** 10.3390/cancers12040788

**Published:** 2020-03-26

**Authors:** William H. Gmeiner, Lance D. Miller, Jeff W. Chou, Anthony Dominijanni, Lysette Mutkus, Frank Marini, Jimmy Ruiz, Travis Dotson, Karl W. Thomas, Graham Parks, Christina R. Bellinger

**Affiliations:** 1 Department of Cancer Biology, Wake Forest School of Medicine, Winston-Salem, NC 27157, USA; ldmiller@wakehealth.edu (L.D.M.); fmarini@wakehealth.edu (F.M.); 2 Comprehensive Cancer Center Wake Forest School of Medicine, Winston-Salem, NC 27157, USA; jchou@wakehealth.edu (J.W.C.); jruiz@wakehealth.edu (J.R.); tdotson@wakehealth.edu (T.D.); kwthomas@wakehealth.edu (K.W.T.); cbelling@wakehealth.edu (C.R.B.); 3 Wake Forest Institute for Regenerative Medicine, Wake Forest School of Medicine, Winston-Salem, NC 27157, USA; adominij@wakehealth.edu (A.D.); lmutkus@wakehealth.edu (L.M.); 4 W.G. (Bill) Hefner Veteran Administration Medical Center, Cancer Center, Salisbury, NC 27157, USA; 5 Department of Pulmonary/Critical Care, Wake Forest School of Medicine, Winston-Salem, NC 27157, USA; 6 Department of Internal Medicine, Wake Forest School of Medicine, Winston-Salem, NC 27157, USA; 7 Department of Pathology, Wake Forest School of Medicine, Winston-Salem, NC 27157, USA; geparks@wakehealth.edu

**Keywords:** SCLC, RNA-Seq, patient-derived organoid, thymidylate synthase, fluoropyrimidine

## Abstract

Chemo-immunotherapy is central to the treatment of small cell lung cancer (SCLC). Despite modest progress made with the addition of immunotherapy, current cytotoxic regimens display minimal survival benefit and new treatments are needed. Thymidylate synthase (TS) is a well-validated anti-cancer drug target, but conventional TS inhibitors display limited clinical efficacy in refractory or recurrent SCLC. We performed RNA-Seq analysis to identify gene expression changes in SCLC biopsy samples to provide mechanistic insight into the potential utility of targeting pyrimidine biosynthesis to treat SCLC. We identified systematic dysregulation of pyrimidine biosynthesis, including elevated *TYMS* expression that likely contributes to the lack of efficacy for current TS inhibitors in SCLC. We also identified E2F1-3 upregulation in SCLC as a potential driver of *TYMS* expression that may contribute to tumor aggressiveness. To test if TS inhibition could be a viable strategy for SCLC treatment, we developed patient-derived organoids (PDOs) from human SCLC biopsy samples and used these to evaluate both conventional fluoropyrimidine drugs (e.g., 5-fluorouracil), platinum-based drugs, and CF10, a novel fluoropyrimidine polymer with enhanced TS inhibition activity. PDOs were relatively resistant to 5-FU and while moderately sensitive to the front-line agent cisplatin, were relatively more sensitive to CF10. Our studies demonstrate dysregulated pyrimidine biosynthesis contributes to drug resistance in SCLC and indicate that a novel approach to target these pathways may improve outcomes.

## 1. Introduction

Small cell lung cancer (SCLC) is a highly lethal lung cancer sub-type with a median survival of only 15–20 months [1]. The majority of SCLC patients present with extensive disease for which the 5-year survival rate is only 2% [2]. Chemotherapy (e.g., cisplatin/carboplatin+etoposide) remains central to SCLC treatment and while highly effective at inducing an initial remission, relapse is nearly inevitable and recurrent disease is unresponsive to further therapy [3,4]. 

Currently, there are no small molecule targeted therapies for SCLC treatment. Unlike NSCLC for which some patients respond to receptor tyrosine kinase (RTK) inhibitors [5], SCLC is not characterized by a reliance on RTK-mediated signaling. Further, SCLC is characterized by near universal disruption of the p53 pathway [6], and upregulation of drug efflux proteins [7,8] that limit the pro-apoptotic and anti-cancer activities of many anti-cancer drugs. SCLCs do, however, express high levels of thymidylate synthase (TS) [9], which is a well-validated target for cancer chemotherapy. High TS activity may indicate SCLC cells are dependent on the de novo nucleotide biosynthetic pathway and thus may be vulnerable to TS inhibitors such as 5-FU [10], tomudex [11], and pemetrexed [12]. However, clinical studies with these agents have not demonstrated strong efficacy in SCLC [10,11,12].

Elevated TS expression is a principal cause of resistance to TS inhibitors, however multiple genes affect overall response. Elevated TS is a cause of both 5-FU-resistance in colon cancer [13] and pemetrexed resistance in NSCLC [14]. Genes other than *TYMS* (which encodes TS) also affect TS inhibitor efficacy. For example, capecitabine, an orally available pro-drug of 5-FU requires activation by thymidine phosphorylase (TP; encoded by *TYMP*), while anti-folate TS inhibitors depend on cell uptake that is mediated by either folate receptors or reduced folate carriers. The efficacy of fluoropyrimidine drugs (FPs) depends on their conversion to FdUMP, the TS inhibitory metabolite [15]. FPs compete with endogenous pyrimidine metabolites for activation and their activity depends on the overall efficiency of de novo pyrimidine biosynthesis and its importance relative to the salvage pathway. Recent studies demonstrate SCLC is highly dependent on de novo pyrimidine biosynthesis for survival [16]. Hence, it is important to consider overall pyrimidine biosynthesis regulation and not solely focus on *TYMS* expression in assessing the potential for targeting TS for SCLC treatment. 

Elevated TS and dysregulated pyrimidine metabolism in SCLC may render conventional TS inhibitors relatively ineffective for SCLC treatment; however, a reliance on de novo Thy biosynthesis represents a highly attractive target for new agents with improved capacity for inhibiting elevated TS. We are developing polymeric FP (e.g., F10 [17]) as a new approach for treating malignancies with elevated TS. We demonstrated significantly increased potency for F10 relative to conventional TS inhibitors [18], including towards cancer cells that express elevated TS [19], and are resistant to 5-FU. The basis for F10’s improved activity is more efficient conversion to FdUMP [17], the TS-inhibitory metabolite of FPs [15]. Further, F10 was equipotent to cancer cells regardless of p53 status [20], which is important for treating p53-null SCLC [21,22]. In addition to strongly inhibiting TS, F10 also causes Topoisomerase 1 cleavage complex (Top1cc) formation. Top1 is the sole target of the camptothecin class of anti-cancer drugs (CPTs), and it is an important target for treating drug-resistant SCLC [23]. Dual targeting of TS/Top1 is a unique mechanism for polymeric FPs [22], with potential therapeutic advantages relative to conventional TS inhibitors and Top1 poisons. To build upon these findings, we recently developed CF10, a 2nd generation polymeric FP that displays improved stability to exonuclease degradation relative to F10, and increased potency to cancer cells.

While elevated *TYMS* is established in SCLC [24,25,26], no comprehensive analysis of genes mediating pyrimidine metabolism was previously reported for SCLC using human clinical samples. Further, the basis for *TYMS* upregulation in SCLC is not known. E2F1 is important for mediating *TYMS* expression in a cell cycle-dependent manner [27]; however, SCLC is almost invariably Rb-null [28]. E2F-family members are implicated in mediating an alternative transcriptional program in the absence of Rb that includes upregulation of genes important for invasion and metastasis [29]. Thus, it is important to consider elevated *TYMS* and dysregulated pyrimidine biosynthesis in the context of altered E2F-family expression and activation of a transcriptional program that contributes to the highly aggressive and metastatic properties of SCLC.

## 2. Results

### 2.1. RNA-Seq Reveals Dysregulated Pyrimidine Biosynthesis in SCLC

**Figure 1:**
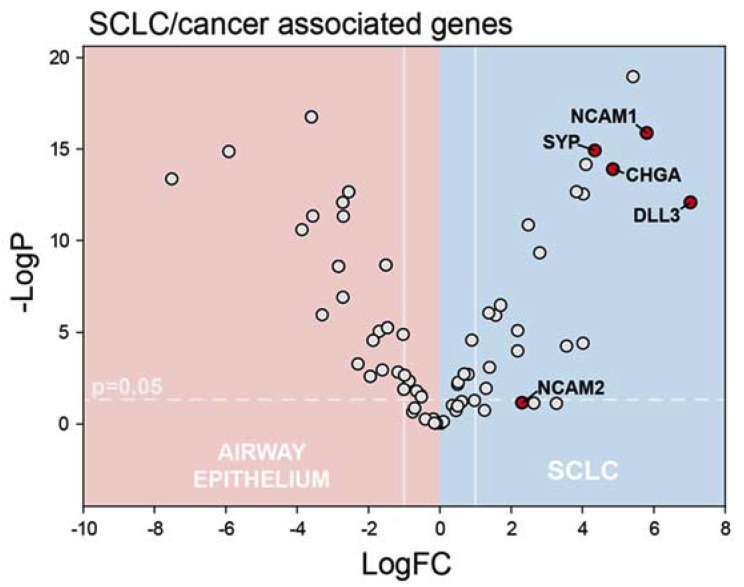
Small cell lung cancer (SCLC) samples displayed elevated expression of genes characteristically expressed at elevated levels in SCLC including *NCAM1*, *SYP*, *CHGA*, and *DLL3* (displayed data are summarized in Supplementary Table S2).

Prior to analyzing RNA-Seq data from SCLC clinical samples (Table S1) for evidence of pyrimidine biosynthesis dysregulation, we evaluated differential expression of genes that are established SCLC biomarkers between the SCLC FNA samples and the brush biopsy normal airway epithelial samples. Chromogranin (CHGA) [30], CD56 (NCAM1) [31], and synaptophysin (SYP) [32] were all significantly upregulated in our SCLC samples (Figure 1). DLL3, which is also characteristically upregulated in SCLC [33], and is under investigation for targeted therapy [34] also was significantly upregulated in our SCLC samples.

**Figure 2:**
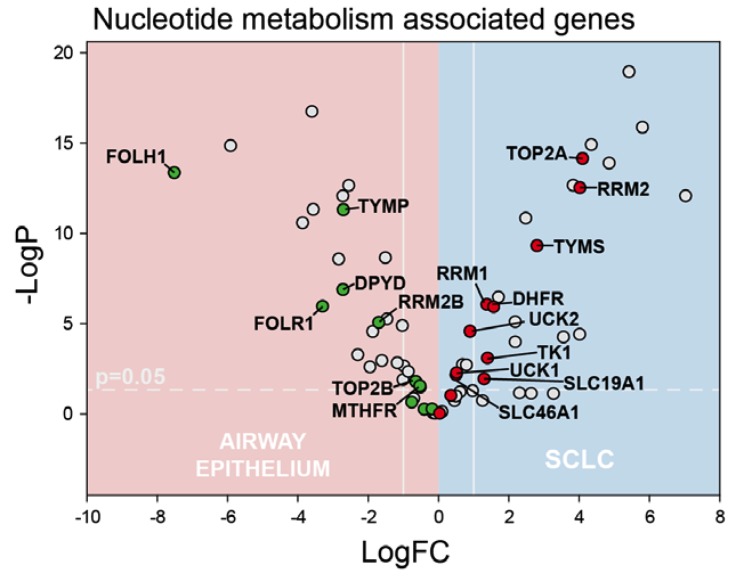
Volcano plot depicting genes important for nucleotide metabolism and pyrimidine biosynthesis that display significantly altered expression in SCLC (blue) relative to non-malignant airway epithelial tissue (pink). Several genes encoding proteins important for de novo Thy biosynthesis are significantly upregulated in SCLC including *TYMS* and *RRM2*.

We then analyzed our RNA-Seq data for systematic differences in gene expression between SCLC and normal airway cells with initial focus on genes that are important for de novo nucleotide biosynthesis (Figure 2), particularly for genes important for activation of fluoropyrimidine drugs. TS is the principal target of FP drugs and TS overexpression is associated with lack of response to 5-FU-based regimens in CRC [13,35,36]. *TYMS* was significantly overexpressed in SCLC relative to normal airway and the extent of overexpression (7.1-fold) is consistent with elevated TS levels that are associated with 5-FU-resistance in colon cancer. Other genes important for de novo Thy biosynthesis were also significantly upregulated in our SCLC samples including *DHFR*, *RRM1*, and *RRM2*. DHFR catalyzes regeneration of the reduced folate co-factor required for TS activity. RRM1 and RRM2 are components of ribonucleotide reductase, which catalyzes enzymatic reduction of ribonucleotides, including UDP, which is a precursor to dUMP, the nucleotide substrate for TS. However, *RRM2B*, which is p53-regulated, was significantly downregulated in SCLC consistent with mutation or deletion of p53 occurring in most SCLC samples. TK, which is important for Thy salvage, is significantly upregulated in SCLC, but the magnitude of *TK1* upregulation is considerably less than for *TYMS* (2.6 vs. 7.1-fold), consistent with a reliance primarily on de novo Thy biosynthesis in SCLC.

*DPYD*, which catalyzes pyrimidine catabolism, was significantly downregulated in our SCLC samples. Increased folate uptake in SCLC was evident in upregulation of *SLC19A1* and *SL46A1*, which encode reduced folate carriers (RFC), while *FOLR1*, which encodes folate receptor α (FRα), was downregulated in SCLC relative to normal airway cells. Most other genes regulating pyrimidine metabolism and FP activity including *UCK1*, *UPP1*, *UPP2*, and *DUT* displayed <2-fold changes in expression in SCLC, a notable exception being *TYMP*, which encodes thymidine phosphorylase (TP), which is significantly downregulated in our SCLC samples. TP catalyzes the reversible conversion of Thy into thymine and it is upregulated in several malignancies and is important for capecitabine activation. Elevated *TYMS* and reduced *TYMP* are consistent with expression of genes regulating pyrimidine metabolism contributing to the lack of efficacy for 5-FU and capecitabine in SCLC treatment. However, this expression profile does not preclude activity for our novel FP polymers in SCLC. FP polymers such as CF10 may be more directly converted to FdUMP and thus display improved activity relative to 5-FU towards malignant cells that express elevated *TYMS*, as we have shown previously in TS-overexpressing colon cancer cells [19]. Further, the activity of FP polymers is likely independent of *TYMP* expression.

### 2.2. Elevated De Novo Thy Biosynthesis Is Associated with E2F1-3 Upregulation 

**Figure 3:**
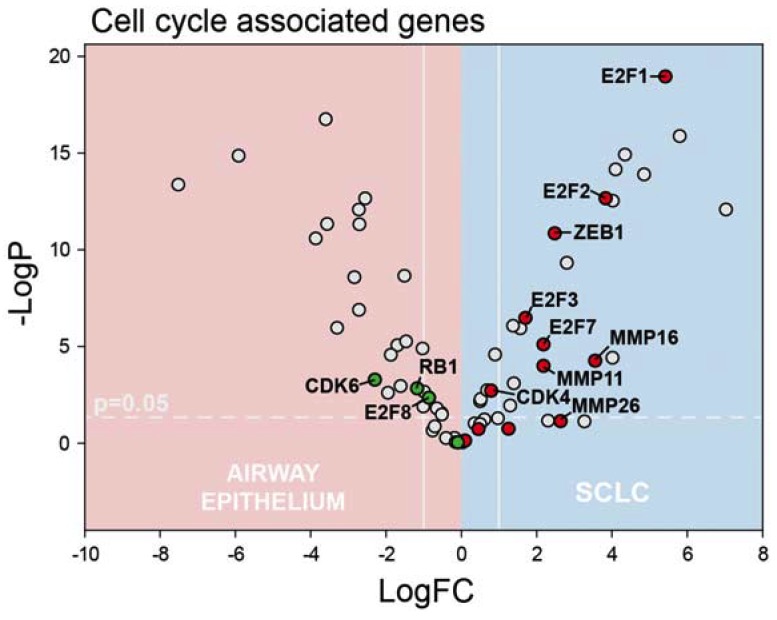
Volcano plot depicting genes involved in cell-cycle regulation. E2F1-3 are transcription factors implicated in upregulating genes important for cell-cycle progression including *TYMS* and *RRM2* and are significantly upregulated in SCLC. In the absence of Rb, E2F-family is involved in upregulating genes important for invasion and metastasis including *ZEB1* and MMPs, including *MMP-11*, *-16*, *-26*.

The genes regulating nucleotide biosynthesis are typically regulated by E2F-family members in a cell-cycle-dependent manner through the phosphorylation of Rb, which in its hypophosphorylated form represses E2F function. Consistent with other studies demonstrating Rb loss in SCLC [37], we detect significantly decreased *RB1* expression in our SCLC samples (Figure 3). CDK4/6, which phosphorylates Rb to drive cell cycle progression and is a therapeutic target in breast cancer and other malignancies, is not upregulated in SCLC. *CDK6* is actually significantly downregulated in our SCLC samples while *CDK4* displays <2-fold increased expression. E2F-family expression was highly variable with *E2F1*, *E2F2*, and *E2F3* significantly upregulated on our SCLC samples while *E2F4*, *E2F5*, and *E2F6* displayed <2-fold changes in expression. *E2F7* was also significantly upregulated in SCLC while *E2F8* was the only E2F family member significantly downregulated in our SCLC samples. It is important to distinguish among E2F family member expression in SCLC because of divergent function with E2F4-5 mainly associated with repressive activity and E2F1 contributing to S-phase entry [38]. Further, overexpression of E2F1 concomitant with Rb-loss is implicated in activating a transcriptional program that drives cancer progression, invasion, and metastasis [29,39,40]. E2F1 is implicated in regulating *Zeb1* and *Zeb2* expression [41], which promotes EMT, and *MMP9* [42], which drives cancer cell invasion. *Zeb1* (but not *Zeb2*), and *MMP9* are significantly upregulated in our SCLC samples (Figure 3). Further, other MMPs not previously implicated as being E2F1-3-regulated including *MMP-11*, *-16*, and *-26* were also significantly upregulated in our SCLC samples. 

### 2.3. Myc-Family Expression and Gene Ontology Analysis

**Figure 4:**
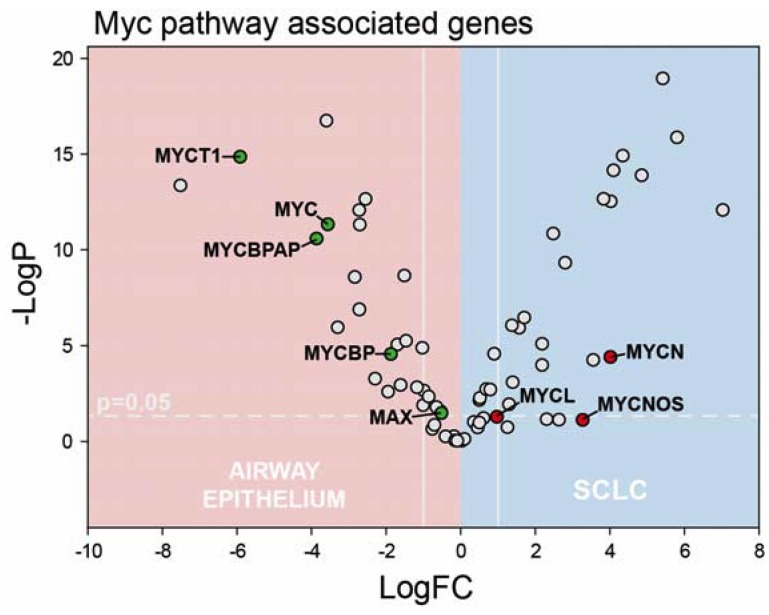
Volcano plot depicting changes in Myc-family expression in SCLC. Only *MYCN* was significantly upregulated and *MYCNOS* was highly expressed. *MYCN* is highly expressed in neuroendocrine sub-type of SCLC consistent with our samples collectively being representative of this sub-type.

Myc-family members are also oncogenic drivers of SCLC. Further, *TYMS* is a Myc-regulated gene and *TYMS* overexpression was previously implicated in compensating for Myc loss and driving proliferation in Myc-knockdown melanoma cells [43]. Each Myc-family member is associated with a different sub-type of SCLC [44], which is predominantly a neuroendocrine malignancy with characteristically high MycN expression [45,46]. *MYCN* was the only Myc-family member significantly overexpressed in our SCLC samples (Figure 4). Interestingly MYCN opposite strand (MYCNOS) [47], a non-coding RNA surrounding the *MYCN* promoter implicated in *MYCN* upregulation in neuroblastoma, was also upregulated in our SCLC samples. Our findings indicate that in addition to E2F1-3 regulation of *TYMS* and genes important for de novo nucleotide biosynthesis in SCLC that *MYCN* could also contribute to their elevated expression. To gain further insight into dysregulation of pathways in SCLC we performed a gene ontology enrichment analysis of our RNA-Seq data using PANTHER. All genes with a log2 fold-change >2.8 were included in our analysis. Among the gene ontology categories identified as significantly upregulated were (Table S3) Cell Division (GO:0051301), mitotic cell cycle process (GO:1903047), and cell cycle process (GO:0022402). Thus, our RNA-Seq data confirmed systematic upregulation of genes that are important components of pathways regulating cell proliferation.

### 2.4. Dysregulated Pyrimidine Biosynthesis Is Associated with 5-FU Resistance, but CF10 Sensitivity

Patient-derived organoids (PDOs) [48] provide a valuable resource for evaluating drug sensitivity ex vivo using clinically-derived tissue samples. Resected tumor tissue is a typical source of material for PDO formation, however, SCLC is a diffuse malignancy and patients rarely undergo surgical resection limiting the utility of this research technique. Further, the small amounts of tissue available from needle aspiration samples obtained to confirm a SCLC diagnosis are insufficient to produce PDOs for drug testing. We therefore investigated whether SCLC FNA samples could be used to first establish PDX tumors in NSG mice and then to produce sufficient tumor volumes for PDO formation. PDX tumors may also be used to directly assess drug response, however, such studies are resource intensive and expensive and generally it is not possible to test more than one or two PDX lines in vivo. In contrast, PDOs can provide information on drug response in samples from multiple patients providing information on the generality of drug response. 

**Figure 5:**
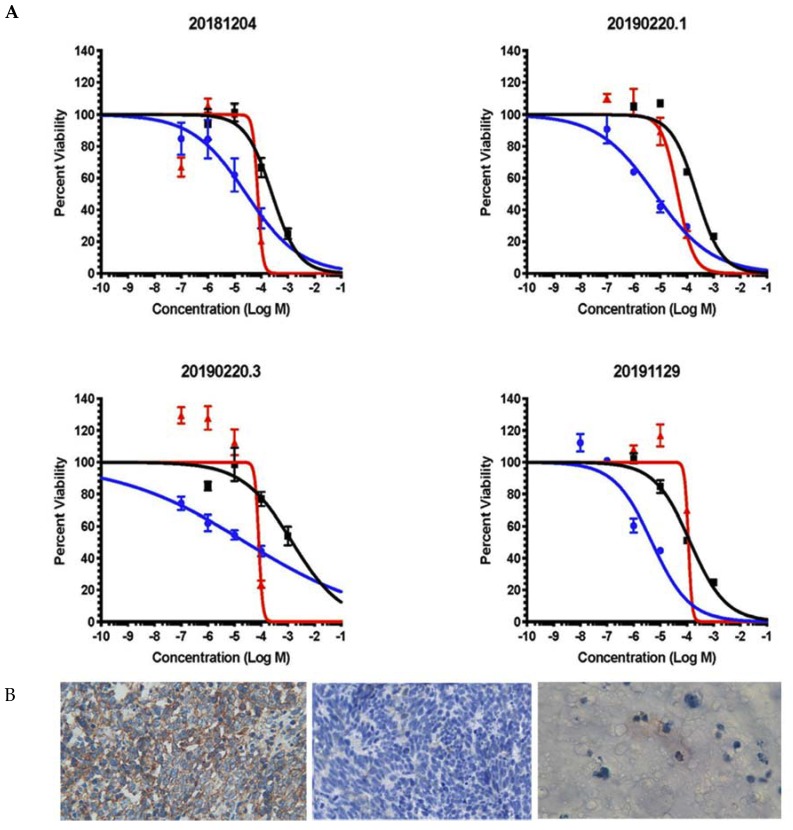
The second-generation polymeric fluoropyrimidine CF10 displays improved potency relative to conventional drugs in SCLC patient-derived organoids (PDOs). (**A**) Dose-response curves for CF10 (blue), cisplatin (red), and 5-FU (black) in SCLC PDOs. PDOs were developed from PDX tumors generated from four different SCLC patient biopsy samples. Aggregate data are shown in Figure S4. Aggregate IC_50_ values: CF10 14.82 μM; Cisplatin 120.7 μM; 5-FU 381.3 μM. CF10 vs. 5-FU: *p* < 0.0001; CF10 vs. cisplatin: *p* < 0.0026. (**B**) CD56 staining for SCLC clinical sample (left; 400×), PDX (middle; 400×), and PDO (right; 600×).

All patient samples were confirmed as SCLC through panel staining including immunostaining for synaptophysin, TTF, and CD56 (Figure S1). We successfully propagated PDX tumors from five FNA samples from confirmed SCLC patients. PDX tumors were expanded to 1000–1500 mm^3^, and then surgically removed. A portion of each tumor was then sectioned, stained with hematoxylin and eosin, and reviewed by a pathologist to confirm propagation of SCLC tumors (Figure S2). SCLC PDX were subject to tumor dissociation to generate PDOs for drug testing ex vivo (Figure S2). We obtained sufficient cell mass from four PDX for PDO ex vivo drug testing (Figure 5). PDX and PDO samples were confirmed for retention of SCLC by (Figure S3) and immunostaining (Figure 5).

We tested cisplatin, 5-FU, and the novel 2nd generation fluoropyrimidine polymer CF10 in our SCLC PDO models (Figure 5). Platinum drugs are used for front-line treatment of SCLC and cisplatin was included as a positive control in our PDO studies. Cisplatin was active at high micromolar concentrations with IC_50_ values ranging from 45.4 to 114.2 μM in PDOs derived from four SCLC patients (Figure 5 and Figure S4). Consistent with our RNA-Seq analysis of SCLC FNA samples that showed elevated expression of genes associated with de novo Thy biosynthesis (Figure 2), PDOs derived from SCLC FNA samples were relatively resistant to 5-FU with IC_50_ values ranging from 0.2564 to 1.475 mM, drug concentrations that could result in intolerable damage to sensitive non-malignant tissues such as gastrointestinal and hematopoietic tissue [22]. 5FU’s lack of efficacy toward SCLC PDOs is consistent with previous clinical trials that demonstrated limited clinical efficacy for 5-FU in SCLC treatment [10]. Since previous studies with TS-overexpressing colon cancer cells showed that elevated *TYMS* was associated with 5-FU resistance [36], but retained sensitivity to polymeric fluoropyrimidines (e.g., F10 [19]), we also tested CF10 in our SCLC PDO models. CF10 is a 2nd generation polymeric FP that shows promising activity in several pre-clinical tumor models. CF10 was much more potent than 5-FU towards PDOs derived from all four SCLC patients tested (4.864–27.57 μM), which is consistent with CF10 being potentially useful for SCLC treatment despite the lack of efficacy for conventional FP drugs (e.g., 5-FU, capecitabine) in treating SCLC. Further, CF10 was consistently more potent than cisplatin in all SCLC PDO models in which both agents were tested, indicating it may be potentially useful for treating relapsed SCLC that is resistant to cisplatin. We went on to test the efficacy of F10 towards one of the PDX lines used for PDO evaluation. F10 was dosed i.p. 2×/week for 4 weeks at approximately 400 mg/kg/dose. This dose was well tolerated and all F10-treated (*n* = 5) and vehicle-treated mice (*n* = 2) gained weight during treatment (Figure S5). Tumor growth curves displayed wide variability for both vehicle- and F10-treated mice and larger studies will be required to determine if F10 and/or CF10 exert anti-tumor activity for SCLC in vivo (Figure S6). 

## 3. Discussion

Chemotherapy is central to SCLC treatment, but current regimens display limited efficacy. Targeted agents that display activity in treating other lung cancer sub-types (e.g., EGFR- and ALK-inhibitors in NSCLC) are relatively ineffective for SCLC because these pathways are generally not dysregulated in SCLC [49]. Further, the spectrum of conventional drugs that are effective for SCLC treatment is also limited, in part, due to near universal inactivation of p53 [6], which limits drug-induced apoptosis. Further, increased expression of drug efflux proteins [7,8] limit accumulation of many anti-cancer drugs in SCLC cells. Thus, new approaches to improve outcomes in SCLC are urgently needed. In light of recent reports that SCLC is highly dependent on de novo pyrimidine biosynthesis for survival [16], approaches targeting pyrimidine biosynthesis are of strong interest.

Targeting de novo nucleotide biosynthesis is a highly effective strategy used to treat multiple malignancies. For example, inhibiting ribonucleotide reductase is effective in treating acute leukemias [50,51], while TS inhibition [15] is central to treatment of gastrointestinal malignancies, including colon [13] and pancreatic cancer [52]. TS inhibition has limited efficacy for SCLC treatment, however. Several previous studies have reported elevated TS levels occur in SCLC cells and tumor tissue [24,25,26], consistent with elevated expression of TS contributing to lack of efficacy for both fluoropyrimidine and anti-folate TS inhibitors. While elevated TS is an important factor affecting drug response, the efficacy of fluoropyrimidine drugs such as 5-FU and capecitabine also depends on efficient conversion to the TS-inhibitory metabolite, FdUMP [15]. Our data show that *TYMP*, which encodes thymidine phosphorylase (TP), is substantially downregulated in our SCLC samples (Figure 2). TP in tumor tissue is important for capecitabine activation and TP together with TK is also important for direct conversion of 5-FU to FdUMP. In colon cancer cells, 5-FU is predominantly converted to ribonucleotides via OPRT [53] (encoded by *UMPS*), and in SCLC the downregulation of *TYMP* coupled with moderately upregulated *UMPS* (log2FC = 0.37; Supplementary Table S2) is consistent with 5-FU also being predominantly converted to ribonucleotides. While 5-FU may induce apoptosis via an RNA-mediated process, this process is p53-dependent [20]. Thus, elevated TS coupled with lack of efficient conversion to FdUMP likely contributes to the lack of efficacy for conventional fluoropyrimidine drugs in SCLC. Further, elevated TS coupled with downregulation of FOLR1 (encoding FRα) and marginally upregulated expression of SLC19A1 and SLC46A1 (encoding reduced folate carriers) likely contributes to a relative lack of efficacy for anti-folate TS inhibitors in SCLC, including pemetrexed and raltitrexed. 

*TYMS* upregulation in SCLC, while contributing to resistance to conventional TS inhibitors, may indicate a reliance on de novo Thy biosynthesis in SCLC that renders this malignancy susceptible to treatment with polymeric fluoropyrimidines, such as CF10, that are more directly converted to FdUMP. We previously showed TS-overexpressing colon cancer cells remained relatively sensitive to F10 [19], a polymeric fluoropyrimidine that showed promising activity in multiple pre-clinical cancer models including AML [22], ALL [54], GBM [55], and prostate cancer [18]. We also showed F10 inhibited TS enzymatic activity at much lower doses than 5-FU [18], consistent with more efficient conversion to FdUMP. F10 cytotoxicity is mediated by a unique mechanism that involves TS inhibition, which stimulates misincorporation of FdUTP into DNA causing topoisomerase 1 cleavage complex formation (Top1cc) [21,22,56]. Top1cc induced by F10 differ from camptothecin because DNA repair occurs under thymineless conditions, which renders repair relatively less effective [57,58]. We recently developed CF10, a 2nd generation polymer with improved nuclease stability relative to F10. The improved stability of CF10 results from inclusion of the non-native nucleoside Cytarabine (AraC) at the 3’-terminus, which reduces exonucleolytic degradation [59]. CF10 displays improved activity relative to F10 in the NCI 60 cell line screen, and greater in vivo anti-tumor activity [60]. CF10 is also being evaluated by the NCI Nanotechnology Characterization Laboratory as a novel, nanoscale material for cancer treatment. Our studies confirm CF10 is much more potent than 5-FU to PDOs developed from SCLC biopsy samples (Figure 5). Further, CF10 was more potent than cisplatin towards SCLC PDOs indicating the potential for CF10 translation to treat this malignancy. For all in vivo studies completed to date, F10 and CF10 have demonstrated greatly reduced systemic toxicities relative to 5-FU [18,22,54,55], which is consistent with reduced conversion to ribonucleotide metabolites that are primarily responsible for 5-FU’s gastrointestinal toxicity [61], which is treated with Urd triacetate [62], consistent with an RNA origin. Our in vivo pilot study demonstrated F10 was well tolerated and all F10-treated mice in our study gained weight indicating higher doses would likely be tolerated (Figure S5). Further testing is needed to establish if FP polymers display anti-tumor activity in SCLC PDX. Use of the more potent analog CF10 in future studies and optimization of dose and schedule may enable establishing in vivo efficacy. 

Since *TYMS*, and other genes important for de novo nucleotide biosynthesis are regulated, in part, by E2F-family members, we also analyzed RNA-Seq data to identify putative drivers of altered gene expression in SCLC that affect the efficacy of TS inhibitors. E2F-family expression was highly variable in SCLC with *E2F1*, *E2F2*, and *E2F3* significantly upregulated. E2F1-3 upregulation in the context of Rb-loss, which is near universal in SCLC, is implicated in upregulating a transcriptional program that drives proliferation and metastasis [29]. Our RNA-Seq data are consistent with dysregulated pyrimidine metabolism being a component of such a pro-metastatic transcriptional program that is upregulated in SCLC. Gene ontology enrichment analysis of our RNA-Seq data identified dysregulation of genes regulating cell division and mitotic cell processes consistent with this interpretation, together with upregulation of transcription factors that drive epithelial-to-mesenchymal transition (EMT; e.g., *Zeb1*), and genes that promote cell invasion (e.g., *MMP9*). Importantly, TS may be an actionable target in the context of this altered transcriptional program and polymeric fluoropyrimidines such as CF10 may be more effective than conventional agents at inhibiting TS, and may result in a positive therapeutic response. 

Of note, a limitation of the current study is the lack of publicly available SCLC RNAseq data profiled together with normal lung epithelium to enable robust confirmation of our current observations. However, we believe the rigorous statistics used in our analysis, that correct for false discovery rate, allow a reliable assessment of gene-pathway relevance in SCLC malignancies. Further work that expands on these observations and seeks to ascertain the relative frequency of pathway involvement in SCLC cases is warranted. 

## 4. Materials and Methods

### 4.1. Samples and Clinical Data

All clinical samples were obtained from consented patients at Wake Forest Baptist Hospital (WFBH) under a protocol approved by the institutional review board (IRB) (IRB Protocol No. IRB00043264). We collected two bronchoscopic fine needle aspirates (FNA) for research purposes from 12 patients who underwent biopsy to confirm a suspected diagnosis of SCLC (Table S1). Patients were offered standard of care treatment. Tumor samples were collected by endobronchial ultrasound-guided transbronchial needle aspiration (EBUS-TBNA) from the primary tumor or proximal lymph nodes. At the time of biopsy, each patient also had mucosal brushings of the right proximal main-stem bronchus to isolate non-malignant airway epithelial cells. Twelve cases of SCLC were confirmed by immunohistochemistry for established SCLC markers including CD56 and chromogranin. We isolated RNA from one FNA research sample and the corresponding brush biopsy sample from each patient. Eight matched pairs were deemed to yield RNA of sufficient quality and quantity to proceed with RNA-Seq analysis. The second FNA research sample was used to form patient-derived xenografts in NOD/SCID mice. PDX tumors were formed from five of the eight patient samples used for RNA-Seq analysis and four of these were used to develop patient-derived organoids (PDO) for drug testing.

### 4.2. EBUS-TBNA

We performed all clinical procedures with patients under deep sedation using a flexible ultrasound bronchoscope (CP-EBUS-XBF-UC260F, Olympus, Tokyo, Japan) and a 21G Vizashot needle. At least three transbronchial needle aspirations were taken at each suspected tumor or nodal station in accordance with diagnostic and staging standards. The initial samples were processed in a standard institutional fashion previously described [63], with preparation of slides for rapid on-site cytological interpretation, as well as collecting material to build a cell-block for delayed pathological evaluation. If rapid on-site cytology reported a high likelihood of tumor from a specified location, a fine needle aspirate (FNA) from the same location was collected and expelled into ice-cold culture media (for PDX generation) or RNAlater (for expression analysis). In addition to the needle aspiration, study participants underwent mucosal brushings of the right proximal main-stem bronchus using a standard, disposable cytology brush. Two brushings were obtained from each subject and stored in RNAlater solution (ThermoFisher).

### 4.3. PDX Generation

This study was approved by the WFU ACUC and all studies were performed in compliance with AALAS guidelines. SCLC tumor biopsy samples for patient derived xenograft (PDX) generation were collected into RPMI media and maintained at 4 °C until used for PDX generation, generally within a few hours. Samples were centrifuged, re-suspended in media, and mixed 1:1 with Matrigel, and then sub-cutaneously injected into the mammary fat pads of 8–10 week old female NSG mice (Charles River). Mice were monitored daily with weight and tumor volumes measured 2×/week. When tumors were 1000–1500 mm^3^, PDX recipients were humanely euthanized by CO_2_ asphyxiation and cervical dislocation and tumors were resected, sectioned and stained to confirm propagation of SCLC, and used for patient-derived organoid (PDO) generation and drug testing. 

### 4.4. PDO Formation and Drug Testing

Tumor organoids [64] were generated using the base material components from HyStem-HP hydrogel kits (ESI-BIO, Alameda, CA), which include a crosslinker, Heprasil, and Gelin-S with the addition of a photoinitiator. The cell suspension was mixed with the hydrogel at a concentration at 100,000 per 10 µL and placed in PDMS-coated wells prior to UV-activated crosslinking. Organoids were grown in DMEM media for 2–3 days prior to a 96 h chemotherapy treatment. Cell viability was analyzed using CellTiter-Glo 3D (Promega) that measures ATP amount in each organoid and calculated compared to non-drugged control. IC_50_ curves were generated using GraphPad Prism. ± SEM. *n* = 3–6 depending on sample.

### 4.5. RNA-Seq

Total RNA was extracted from tumor specimens and brush biopsies using the miRNAeasy Micro Kit with Qiazol (Qiagen). Samples were assessed for quality by electrophoretic tracing (Agilent Bioanalyzer) and libraries were generated using the Illumina TruSeq Stranded Total RNA kit with Ribo-Zero rRNA depletion. Briefly, 100–500 ng of total RNA was depleted of rRNA, fragmented, and reverse-transcribed to double-stranded cDNA, then purified using AMPure XP magnetic beads. After subsequent cDNA end repair and 3′ adenylation, Illumina sequencing adaptors were ligated to fragment ends, and the libraries were pre-amplified with PCR. Library size distributions were inspected for quality using an Agilent 2100 Bioanalyzer. Library quantity was measured using the Qubit 3.0 (Thermo Fisher, USA). Indexed libraries were then pooled and sequenced to a target read depth of 30 M reads per library using 75 bp single-end sequencing on an Illumina NextSeq 500 high-output flow cell. Sample read depths ranged from 22 to 40 M. For each sample, approximately 80% of sequences achieved >Q30 Phred quality scores (FASTQC analysis, Babraham Bioinformatics). Adapter contamination was cleaned with Trimmomatic [65]. Reads were aligned to the reference human genome GRCh38 using the STAR sequence aligner [66], and gene counts determined using featureCounts software [67]. Differentially expressed genes were identified by negative binomial modeling using DESeq2 [68]. This work was performed by the Cancer Genomics Shared Resource of the Wake Forest Baptist Comprehensive Cancer Center.

## 5. Conclusions

SCLC remains one of the most intransigent of malignancies. Our studies provide new insights into how dysregulated expression of genes important for nucleoside metabolism contribute to the lack of efficacy for conventional fluoropyrimidine drugs (5-FU, capecitabine) in SCLC treatment. In particular, we show that not only does elevated *TYMS* contribute to resistance, but downregulated *TYMP* may contribute to fluoropyrimidine resistance by limiting conversion to the active metabolite, FdUMP. Expression of *TYMS* and other genes important for de novo nucleotide biosynthesis is regulated by E2F-family members and our RNA-Seq data demonstrate E2F1-3 are significantly upregulated in SCLC. Several genes implicated in invasion and metastasis (e.g., *MMP9*, *Zeb1*) are under E2F-family regulation under conditions where Rb is lost, such as is in SCLC. Our RNA-Seq data are consistent with alterations in nucleotide metabolism also being a component of such a pro-metastatic transcriptional program. We also identified previously unreported genes including *MMP-11*, -*16*, and -*26* that are significantly upregulated in SCLC and which may be regulated by E2F-family transcription factors and contribute to the strong propensity for SCLC to metastasize.

TS is a well-validated target for cancer treatment and TS inhibitors are central components of many chemotherapy regimens used to treat a number of malignancies, including colon cancer. Elevated TS could indicate a reliance on de novo Thy biosynthesis in SCLC that renders TS an actionable target. Thus, new drugs that more efficiently inhibit TS and are less vulnerable to dysregulated pyrimidine metabolism may be effective for SCLC treatment. Our studies with PDOs derived from SCLC biopsy samples demonstrate that CF10 is significantly more effective than 5-FU towards all patient samples tested. CF10 was also more potent than cisplatin indicating therapeutic potential for SCLC treatment, possibly including activity in cisplatin-resistant disease.

## 6. Patents

Wake Innovations has filed a patent application on CF10 for treatment of colorectal cancer, which is pending.

## Supplementary Materials

The following are available online at https://www.mdpi.com/2072-6694/12/4/788/s1, Table S1: Patient characteristics for SCLC clinical samples, Table S2: Summary of gene expression differences between SCLC and non-malignant airway tissue, Table S3: Summary of Geneo Ontology differences from Panther analysis, Figure S1: Images of panel staining used to confirm SCLC in clinical samples. Figure S2: Diagram of flow of clinical samples to create PDX and PDO samples for these studies. Figure S3: H&E-stained section of PDX tumor developed from SCLC patient sample, Figure S4: Aggregate dose response curves for CF10, cisplatin, and 5-FU in SCLC patient-derived organoids (PDOs). Figure S5: Representative data from PDX study showing response to CF10 in vivo.
