# Supplementary material for: Dysregulated Pyrimidine Biosynthesis Contributes to 5-FU Resistance in SCLC Patient-Derived Organoids but Response to a Novel Polymeric Fluoropyrimidine, CF10
Source: Cancers (Basel). 2020 Mar 26;12(4):788. doi: 10.3390/cancers12040788 (PMC7226016; doi:10.3390/cancers12040788)
Supplement: Supplementary file 1 [file cancers-12-00788-s001.pdf]

cancers

MDPI

Article

Dysregulated Pyrimidine Biosynthesis Contributes to 5-FU Resistance in SCLC Patient-Derived Organoids but Response to a Novel Polymeric Fluoropyrimidine, CF10

William H. Gmeiner, Lance D. Miller, Jeff W. Chou, Anthony Dominijanni, Lysette Mutkus, Frank Marini, Jimmy Ruiz, Travis Dotson, Karl W. Thomas, Graham Parks and Christina R. Bellinger

Supplementary Information

Table S1. Patient characteristics for SCLC clinical samples used in studies.

Age	Weight	Height	Race	Ethnicity	Primary	History of Smoking - Pack Years
54	141	67	White or Caucasian	Not Hispanic or Latino	Lung	117
53	178.5	71	White or Caucasian	Not Hispanic or Latino	Lung	~30
56	128	61	White or Caucasian	Not Hispanic or Latino	Lung	44
66	239	71.5	White or Caucasian	Not Hispanic or Latino	Lung	20
69	203	67	White or Caucasian	Not Hispanic or Latino	Lung	50
61	186	71	White or Caucasian	Not Hispanic or Latino	Lung	98
54	211	69	Black or African Ame	Not Hispanic or Latino	Lung	38
61	229	72	White or Caucasian	Not Hispanic or Latino	Lung	70 - 100
61	134	62	White or Caucasian	Not Hispanic or Latino	Lung	50 plus years
54	198.8	70	White or Caucasian	Not Hispanic or Latino	Lung	60
67	208	67	White or Caucasian	Not Hispanic or Latino	Lung	12
53	121		White or Caucasian	Hispanic or Latino	Lung	Unknow Smoke 0.5 packs a day.

Table S2. Summary of gene expression differences between SCLC and non-malignant airway tissue included in our analysis.

Gene	Log2FC (Fl	pvalue	padj
CHGA	4.87	1.63E-16	1.40E-14
NCAM1	5.82	9.63E-19	1.45E-16
NCAM2	2.32	2.87E-02	7.55E-02
SYP	4.36	1.17E-17	1.33E-15
DLL3	7.05	1.72E-14	9.00E-13
RB1	-1.16	3.43E-04	1.60E-03
CDK4	0.81	4.69E-04	2.11E-03
CDK6	-2.28	1.06E-04	5.66E-04
E2F1	5.43	3.40E-22	1.21E-19
E2F2	3.85	3.87E-15	2.38E-13
E2F3	1.72	2.77E-08	3.65E-07
E2F4	0.12	5.25E-01	8.05E-01
E2F5	-0.07	7.59E-01	9.85E-01
E2F6	0.47	9.20E-02	2.02E-01
E2F7	2.20	9.46E-07	8.61E-06
E2F8	-0.84	1.22E-03	4.88E-03
MMP9	1.27	8.91E-02	1.97E-01
MMP11	2.20	1.66E-05	1.10E-04
MMP16	3.57	8.34E-06	5.95E-05
MMP26	2.65	3.03E-02	7.91E-02
ZEB1	2.50	4.02E-13	1.55E-11
MAX	-0.50	1.20E-02	3.58E-02
MYC	-3.55	1.19E-13	5.10E-12
MYCL	0.98	2.02E-02	5.59E-02
MYCN	4.03	5.64E-06	4.20E-05
MYCNOS	3.29	3.16E-02	8.20E-02
MYCBPAP	-3.85	7.87E-13	2.86E-11
MYCBP	-1.86	3.78E-06	2.95E-05
MYCT1	-5.90	1.37E-17	1.51E-15

Gene	Log2FC (Fl	pvalue	padj
DHFR	1.58	1.10E-07	1.26E-06
DPYD	-2.70	9.41E-09	1.38E-07
DUT	0.04	8.31E-01	9.85E-01
FOLH1	-7.50	6.24E-16	4.69E-14
FOLR1	-3.28	1.03E-07	1.18E-06
MTHFR	-0.51	9.84E-03	3.00E-02
RRM1	1.39	7.81E-08	9.28E-07
RRM2	4.04	5.48E-15	3.24E-13
RRM2B	-1.68	1.04E-06	9.40E-06
SLC19A1	1.31	3.56E-03	1.24E-02
SLC46A1	0.52	1.94E-03	7.33E-03
TOP1	-0.17	3.37E-01	5.78E-01
TOP2A	4.11	8.37E-17	7.66E-15
TOP2B	-0.63	5.20E-03	1.72E-02
TK1	1.41	1.73E-04	8.74E-04
TYMS	2.82	1.97E-11	5.20E-10
TYMP	-2.69	5.26E-12	5.26E-12
UCK1	0.53	1.45E-03	5.69E-03
UCK2	0.91	3.59E-06	2.81E-05
UMPS	0.37	4.14E-02	1.03E-01
UPP1	-0.75	1.13E-01	2.39E-01
UPP2	-0.39	3.48E-01	5.92E-01

Table S3. Gene ontology pathways upregulated in SCLC (based on genes >2.8-fold upregulated).

	Reflist	Input	Expected over		Fold enrich	*p*-value	FDR
cell division (GO:0051301)	495	60	26.64	+	2.25	4.27E-08	5.03E-06
mitotic cell cycle process (GO:1903047)	600	70	32.29	+	2.17	1.80E-08	2.37E-06
cell cycle process (GO:0022402)	979	92	52.69	+	1.75	1.05E-06	8.97E-05

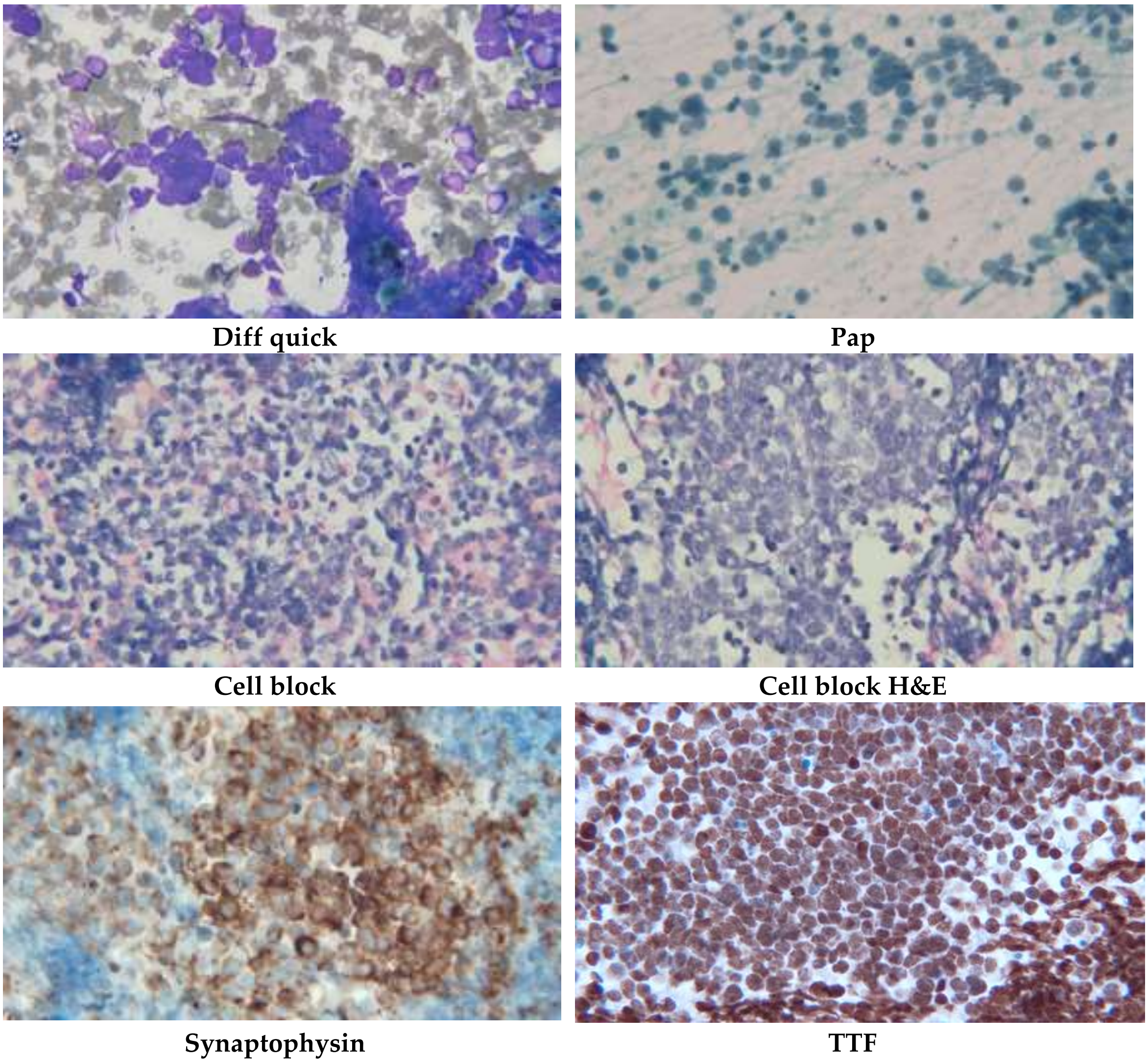

Figure S1. Images (400×) of slides from one of the patients included in this study. Indicated staining was used to confirm SCLC. Diff quick: Malignant cells with high nuclear to cytoplasmic ratios, hyperchromatic nuclei, and characteristic crush artifact. Pap: Alcohol-fixed preparations demonstrate nuclei with characteristic speckled chromatin consistent with neuroendocrine differentiation. Cell block: H and E slides of the cell block show a high grade malignant neoplasm with necrosis. Immunostains: Malignant cells are strongly positive for TTF-1 and synaptophysin confirming the diagnosis of small cell carcinoma.

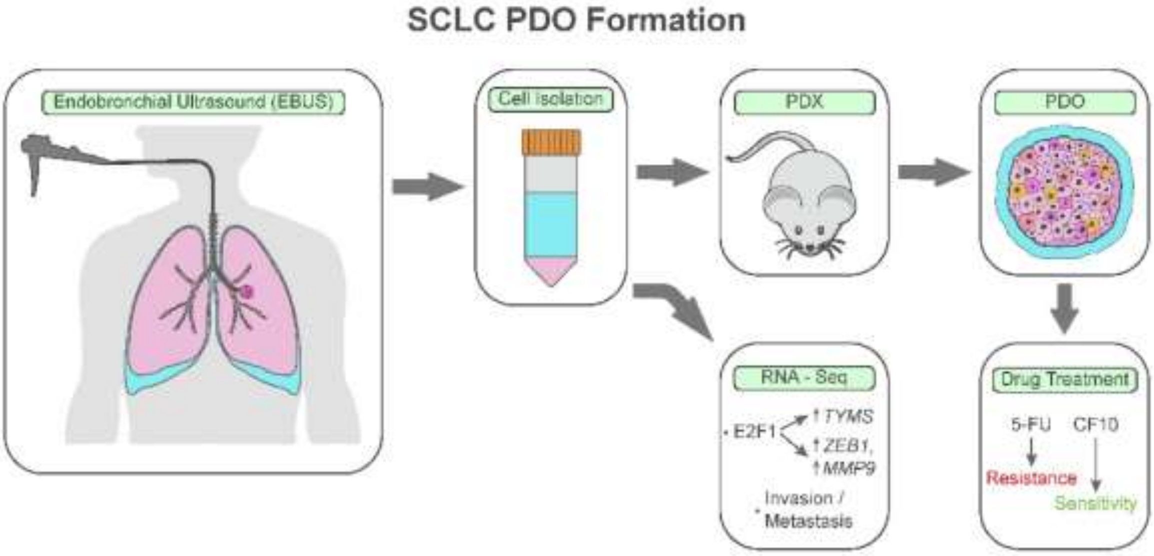

Figure S2. Diagram indicating the flow of materials in these studies. SCLC tissue was obtained via EBUS and underwent cell isolation to enrich in SCLC cells which were then used for PDX generation and RNA-Seq. PDX were used as a source of SCLC cells for PDO formation which were then used for drug testing.

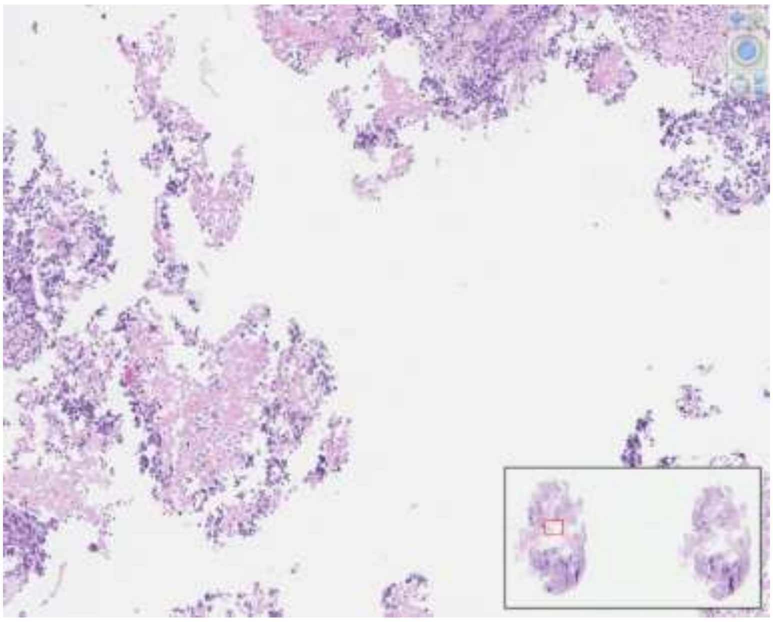

Figure S3. H&E-stained section of PDX tumor developed from SCLC patient sample. Slide was reviewed by a pathologist and determined to be consistent with SCLC.

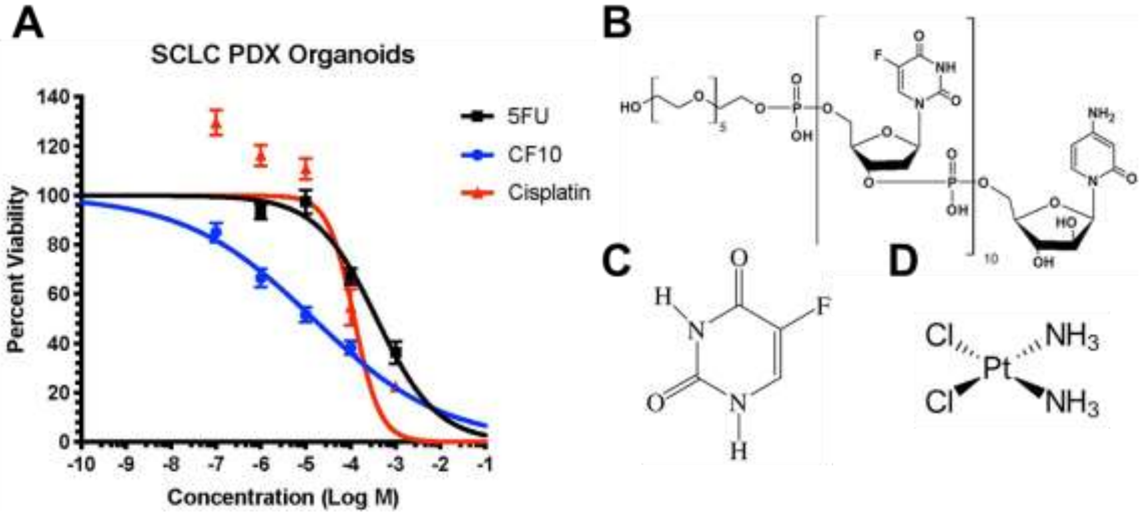

Figure S4. Aggregate dose-response curves for CF10 (blue), cisplatin (red), and 5-FU (black) from SCLC patient-derived organoids (PDOs) developed from four PDX tumors that were generated from four different SCLC patient samples. Dose response curves from the individual patient samples are displayed in Figure 5 while the average of the fours is displayed in (**A**). (**B**–**D**) Structures of CF10, 5-FU, and cisplatin.

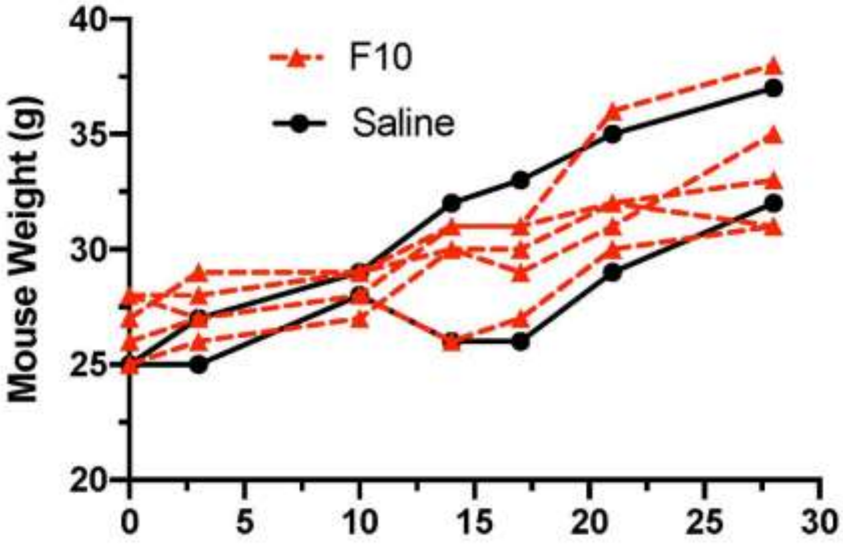

Figure S5. F10 does not induce weight-loss in NSG mice. Mice were treated with either F10 (*n* = 5; i.p. 2×/week for 4 weeks at approximately 400 mg/kg/dose) or saline (*n* = 2). Previous studies with FP polymers indicated that actual drug concentrations as determined based on UV absorbance were up to 50% water hence actual F10 dose administered in this study is estimated as 200 mg/kg/dose. Higher doses of F10 and CF10 may be tolerated and may exert improved anti-tumor activity.

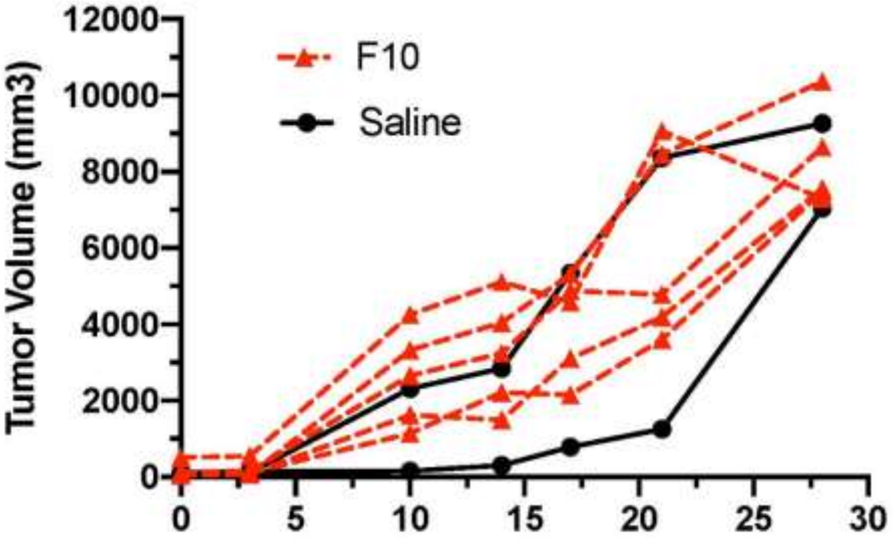

Figure S6. Time-dependent changes in tumor volume for one SCLC PDX model. Mice were treated with either F10 (n = 5; i.p. 2×/week for 4 weeks at approximately 400 mg/kg/dose) or saline (n = 2). Considerable range in SCLC PDX tumor growth rates occur for both vehicle- and F10-treated mice. Additional testing is needed to determine if FP polymers are effective at reducing SCLC tumor growth rates in vivo.
